# High-sensitivity nanophotonic sensors with passive trapping of analyte molecules in hot spots

**DOI:** 10.1038/s41377-020-00449-7

**Published:** 2021-01-05

**Authors:** Xianglong Miao, Lingyue Yan, Yun Wu, Peter Q. Liu

**Affiliations:** 1 Department of Electrical Engineering, University at Buffalo, The State University of New York, Buffalo, NY 14260 USA; 2 Department of Biomedical Engineering, University at Buffalo, The State University of New York, Buffalo, NY 14260 USA

**Keywords:** Optical sensors, Imaging and sensing

## Abstract

Nanophotonic resonators can confine light to deep-subwavelength volumes with highly enhanced near-field intensity and therefore are widely used for surface-enhanced infrared absorption spectroscopy in various molecular sensing applications. The enhanced signal is mainly contributed by molecules in photonic hot spots, which are regions of a nanophotonic structure with high-field intensity. Therefore, delivery of the majority of, if not all, analyte molecules to hot spots is crucial for fully utilizing the sensing capability of an optical sensor. However, for most optical sensors, simple and straightforward methods of introducing an aqueous analyte to the device, such as applying droplets or spin-coating, cannot achieve targeted delivery of analyte molecules to hot spots. Instead, analyte molecules are usually distributed across the entire device surface, so the majority of the molecules do not experience enhanced field intensity. Here, we present a nanophotonic sensor design with passive molecule trapping functionality. When an analyte solution droplet is introduced to the sensor surface and gradually evaporates, the device structure can effectively trap most precipitated analyte molecules in its hot spots, significantly enhancing the sensor spectral response and sensitivity performance. Specifically, our sensors produce a reflection change of a few percentage points in response to trace amounts of the amino-acid proline or glucose precipitate with a picogram-level mass, which is significantly less than the mass of a molecular monolayer covering the same measurement area. The demonstrated strategy for designing optical sensor structures may also be applied to sensing nano-particles such as exosomes, viruses, and quantum dots.

## Photonic sensors: trapping analytes by digging into nano-trenches

An innovative sensor can make it easier to use infrared light for label-free, picogram-scale detection of amino acids and other molecules. Photonic sensors avoid the need for large quantities of analytes by using nanoscale gap structures to trap targets and subject them to ultrahigh optical fields. Peter Liu and colleagues at the State University of New York at Buffalo in the United States have developed a microchip that naturally drives molecules into nanoscale gap regions. The team fabricated arrays where aluminum ribbons overhang narrower strips of insulating crystals. When a drop of test solution is placed onto the chip and left to evaporate, surface tension creates concave profiles that concentrate molecules under the aluminum layers. The resonant frequencies within this device’s “nano-trenches” can be tuned to specific molecular targets by tweaking the array’s geometric parameters.

## Introduction

Infrared absorption spectroscopy is a powerful tool for label-free identification of molecules through their vibrational fingerprints and is widely used in scientific research and industrial applications such as biomolecular analysis^1,2^, process monitoring^3,4^, and pollutant detection^5,6^. Nevertheless, molecular vibrational absorption is intrinsically weak owing to its orders-of-magnitude smaller dipole moment than that of infrared wavelengths; hence, a large number of molecules are needed to achieve detectable absorption of incident infrared light. An effective approach to overcome this limitation is exploiting the strong optical near-fields of nanophotonic structures to significantly enhance molecular absorption, which is proportional to the field intensity experienced by the molecules^7,8^. The recent development of a wide range of nanophotonic structures and devices constitutes a suitable platform for developing molecular sensors based on surface-enhanced infrared absorption (SEIRA) spectroscopy. A variety of photonic structures have been employed for SEIRA sensing, such as nano-rods^9,10^, split-ring resonators^11^, colloidal nano-particles^12–15^, and metal-insulator-metal type structures^16–18^. To enhance sensitivity, structures with nanometric gaps for achieving ultrahigh field confinement and enhancement were demonstrated^17,19–22^. For example, sensors based on individual bowtie antennas with a sub-3 nm gap were able to resolve vibrational signals from a few hundred molecules in the gaps^19^. Regardless of specific photonic structure designs, regions of highly enhanced near-field intensity, also referred to as hot spots, only occupy a small fraction of the total surface area of a photonic structure. Surface-enhanced molecular absorption is only significant in these hot spots. In general, higher field enhancement is typically associated with smaller hot spots.

To conduct SEIRA sensing, the analyte is usually coated over the entire sensor surface using methods such as the self-assembly of molecular layers^9,11,23^, spin coating^20,24^, DNA/protein immobilization, or the physical adsorption of biomolecules^21,25^. A common drawback of these convenient methods is that analyte molecules are distributed across the entire sensor surface. Therefore, only a small percentage of all analyte molecules are delivered to the sensor hot spots that produce an enhanced molecular vibrational absorption signal, whereas the majority of the analyte molecules are outside of the hot spots and do not contribute significantly to the overall sensing signal. This issue can be a major limiting factor for the sensitivity performance of SEIRA sensors as well as optical sensors in general but has not been adequately addressed in most sensor designs. Enhancing the near-field intensity is an important design strategy that has been constantly improved by employing structures with ever-smaller hot spots; however, this approach does not necessarily lead to overall sensitivity improvement alone, as reducing the sizes of hot spots typically results in a smaller amount of analyte molecules delivered to the hot spots. Therefore, developing effective approaches for targeted and efficient delivery of analyte molecules to hot spots is perhaps an equally important aspect for optimizing optical sensor performance. One of the explored approaches is to build micro- and nano-fluidic structures, such as nano-pores^26^ or nano-gaps^27,28^, to guide analyte solutions to hot spots. Although, in principle, this approach can ensure that all analyte molecules pass through the hot spots, as the molecules are still distributed in the solution and there is no concentrating mechanism, at any moment, the number of molecules in the hot spots is determined by the solution concentration. Active trapping mechanisms such as dielectrophoresis^29,30^, optical trapping^31–33^, and micro-bubble trapping^34^ are promising ways to concentrate and deliver nano-objects and large biomolecules (e.g., proteins and DNA). However, these trapping mechanisms require external energy sources such as a laser or an applied voltage and are not suitable for relatively small molecules. Super-hydrophobic artificial surfaces consisting of arrays of micro-pillars have been employed for passively confining large biomolecules in diluted solutions to nanophotonic structures, which led to impressive sensitivity performance^35,36^. However, this approach has not achieved targeted delivery of analyte molecules only to the hot spots of nanophotonic structures.

Here, we present a nanophotonic sensor design that employs hot spot structures that can passively retain and concentrate an analyte solution as it evaporates and eventually traps precipitated analyte molecules inside the hot spots, significantly enhancing its SEIRA sensitivity. We demonstrated this passive trapping functionality using several molecular species (l-proline, d-glucose, and sodium chloride) as well as nano-particles (liposomes). These SEIRA sensors reliably produce clear spectral responses to the molecular vibrational absorption associated with picogram-level analyte precipitates.

## Results

### Device structure design

**Fig. 1:**
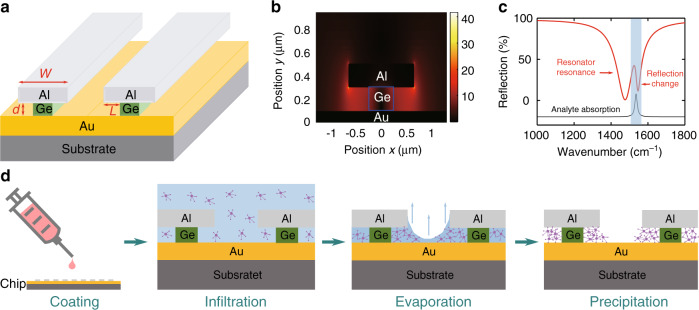
**a** Schematic of the sensor structure. The parameters *w*, *d*, and *L* represent the width of Al ribbons, the height of Ge ribbons and the width of nano-trenches, respectively. **b** Simulated electric near-field distribution of an exemplary resonator design with *d* = 200 nm, *w* = 1.4 μm, and *L* = 400 nm. **c** Reflection spectrum (red curve) of the resonators in **b** with the nano-trenches filled with an “imaginary” molecular species that have a single absorption line at ~1550 cm^−1^ (black curve). **d** Schematics depicting the underlying process of the molecule trapping functionality of the nano-trench structures

A schematic of our device structure is shown in Fig. 1a. The designed optical resonators have a metal-insulator-metal type structure, which consists of a periodic array of Al ribbons on top of Ge ribbons, with an Au back reflector underneath. We chose Ge mainly because it has relatively low optical loss in the target spectral region and can be deposited on metal films using the electron beam evaporator in our cleanroom facility, whereas in principle, it can be replaced by any other material with low optical loss, such as Si. The Ge ribbons are narrower than the Al ribbons by hundreds of nm; therefore, nano-trenches are formed on both sides of each Ge ribbon. Such a structure supports a resonance mode that can be excited by incident light polarized perpendicular to the Al ribbons, and the resonance frequency can be designed to target specific absorption lines of a molecular species by tailoring the geometrical parameters such as the Al ribbon width *w*, the nano-trench width *L*, and the Ge ribbon height *d*. Figure 1b shows the simulated distribution of electric near-field enhancement of an exemplary design at its resonance frequency. The electric field is mostly confined inside the nano-trenches, and the highest electric field enhancement can reach over 40, which corresponds to an intensity enhancement of more than three orders of magnitude. Therefore, these nano-trenches are the hot spots of our resonator design. Molecules located inside the nano-trenches experience high-field intensity enhancement, which in turn leads to an enhanced spectral response to molecular vibrational absorption. As an example, Fig. 1c shows the reflection spectrum of a resonator design with its nano-trenches filled with an “imaginary” molecular species that have a single absorption line (black curve) near the resonance frequency of the resonator. The reflection spectrum clearly exhibits a Fano-resonance type feature near the molecular absorption line as a result of the molecular vibrational mode interfering constructively or destructively with the enhanced electric field in the nano-trenches^37^. In addition to achieving a large-field enhancement, another key advantage of incorporating these nano-trenches in our resonator design is that molecules in a low-concentration analyte solution can be passively trapped inside the nano-trenches as the analyte solution gradually evaporates. Figure 1d illustrates such a passive molecule trapping process. A droplet of analyte solution can be introduced to the device surface using a micro-pipette. The analyte solution covers the entire resonator array and infiltrates into the nano-trenches. Subsequently, the solvent of the analyte solution gradually evaporates, and the solution top surface gradually lowers, whereas the concentration of the solution increases. When the solution top surface is below the Al ribbon top surface, a concave profile of the solution surface forms between neighboring Al ribbons owing to surface tension, and the edge of the solution is pinned at the side of the Al ribbons^38,39^. As the solvent evaporates further, the concave part ruptures near its center, the solution is retained inside the nano-trenches and further concentrates, and eventually, most of the analyte molecules precipitate inside and near the edges of the nano-trenches as the solvent completely evaporates.

**Fig. 2:**
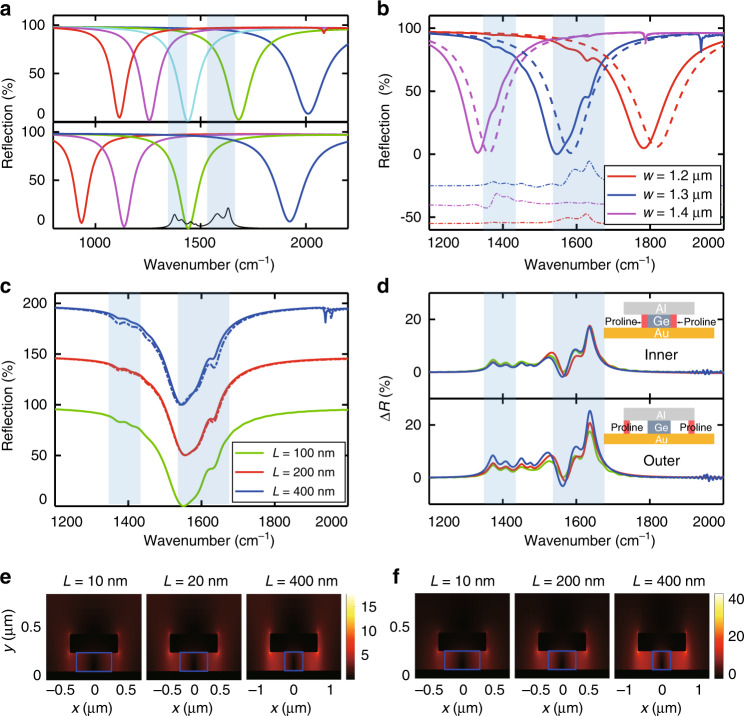
Numerical simulation results. **a** Top: reflection spectra of resonator designs with Al ribbon widths ranging from 800 nm to 1.4 μm. The nano-trench width is 200 nm. Bottom: reflection spectra of resonator designs with nano-trench widths ranging from 0 to 300 nm. The Al ribbon width is 1 μm. The black curve corresponds to the proline absorption spectrum, and the shaded regions mark the main proline absorption lines. **b** Reflection spectra of three resonator designs with the specified Al ribbon widths and a fixed nano-trench width of 400 nm before (dashed curves) and after (solid curves) the inner side of the nano-trenches is filled with 100 nm wide proline. The dot-dashed curves are the differential reflection spectra of these resonators due to the addition of proline. **c** Reflection spectra of three resonator designs with the specified nano-trench widths, with 100 nm wide proline filling either the inner side (solid curves) or the outer side (dashed curves) of the nano-trenches. **d** Extracted differential reflection spectra of the resonator designs in **c** due to proline filling the inner sider (top) and the outer side (bottom) of the nano-trenches. The insets show where the proline is added. It should be noted that for the design with *L* = 100 nm, the 100 nm wide proline fills the nano-trenches completely, so there is no distinction between the inner side and the outer side in this case. **e** and **f** Simulated electric field distributions of the resonator designs in **c** at the frequencies of two proline absorption lines, i.e., 1453 cm^−1^ and 1630 cm^−1^, respectively. The blue solid lines represent the Ge ribbons

Numerical simulations were conducted by using the finite difference time domain (FDTD) method (see “Materials and methods”) to investigate how the Al ribbon width (*w*) and the nano-trench width (*L*) affect the optical response as well as sensing performance. Figure 2a shows the reflection spectra of several resonator designs with different geometrical parameters. The top panel corresponds to designs with different *w* values (ranging from 800 nm to 1.4 μm) and a fixed *L* = 200 nm, whereas the bottom panel corresponds to designs with different *L* values (ranging from 0 to 300 nm) and a fixed *w* = 1 μm. The height of the Ge ribbon is fixed at 200 nm in these designs. These design parameters can be reliably realized using standard nanofabrication processes, and the corresponding wide spectral tuning range covers infrared vibrational absorption bands of a broad variety of molecular species. Furthermore, we also avoided any design parameter that may weaken the structural robustness of the devices so that the devices can withstand the processes for introducing and removing the target analyte and be used for repeated measurements. As an example for demonstrating the sensing capability of these resonator designs, we chose l-proline, an amino acid, as the analyte molecule, which has several vibrational absorption lines in the spectral range between 1400 cm^−1^ and 1700 cm^−1^ (see the black solid line and shaded regions in Fig. 2a). Figure 2b shows the simulated reflection spectra of three resonator designs with the specified Al ribbon widths and a fixed nano-trench width *L* = 400 nm before (dashed curves) and after (solid curves) placing 100 nm wide proline on the inner side of the nano-trenches (i.e., adjacent to the Ge ribbon sidewall). The resonance frequency of the resonators was tuned across several proline absorption lines in this spectral range. The extracted reflection changes due to the added proline were plotted as dot-dashed curves. As expected, the spectral response to a specific proline absorption line is the strongest when the resonance frequency of a resonator matches that absorption line. We further investigated the influence of the nano-trench width on the sensing performance in Fig. 2c, which shows three resonator designs with different *L* values (as well as different *w* values so that their resonance frequencies are similar), with 100 nm wide proline filling either the inner side (solid curves) or the outer side (dashed curves) of the nano-trenches. Note that for *L* = 100 nm, the 100 nm wide proline simply filled the entire nano-trench. The extracted spectral changes owing to the added proline in the nano-trenches are plotted in Fig. 2d. Comparing the different spectra in Fig. 2c, d, we found that the resonator designs with relatively wide nano-trenches produced a stronger spectral response, and the spectral response was larger when the same amount of proline was located on the outer side of the nano-trenches than on the inner side. These observations can be explained by the electric field distributions in these nano-trenches. The electric near-field distributions of the three resonator designs at frequencies of 1452 cm^−1^ and 1630 cm^−1^ are shown in Fig. 2e, 2f, respectively. It can be clearly seen that overall, the designs with wider nano-trenches have higher field enhancement, and the field enhancement in a nano-trench is always larger on its outer side than on its inner side.

### Device fabrication

**Fig. 3:**
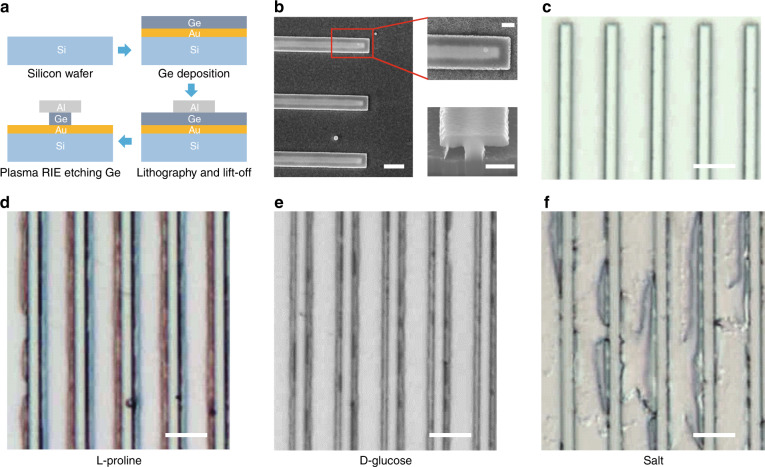
**a** Schematics of the device fabrication process. **b** SEM images of the fabricated devices taken at an electron accelerating voltage of 20 kV. The left and upper-right images are top views of a device structure, in which the Al ribbons, the underlying nano-trenches (darker gray color) and Ge ribbons (brighter gray color) are clearly visible. The lower right image is the cross-sectional view of a resonator structure cut using FIB. The scale bar in the left image is 2 μm, and the scale bars in the right two images are 600 nm. **c**–**f** Optical microscope images of **c** the bare device, **d** the device with l-proline precipitate, **e** the device with d-glucose precipitate, and **f** the device with sodium chloride precipitate. The scale bars in **c**–**f** are all 5 μm

Figure 3a illustrates the fabrication process to realize the designed device structures. Compared with nanophotonic sensors employing nanometric gaps or super-hydrophobic artificial surfaces, our device designs are easier to fabricate. The entire fabrication process involves only one lithography step and one dry etching step, which is similar to typical metal-insulator-metal photonic structures without nano-trenches (see fabrication details in “Materials and methods”). Each fabricated resonator array occupies an area of 300 μm by 300 μm. Figure 3b shows scanning electron microscopy (SEM) images of the fabricated devices. In the top-view images of the structure (the left and upper-right panels), the bright edges of individual Al ribbons can be clearly seen. Adjacent to the bright Al ribbon edges are stripes exhibiting a dark gray color, which is in stark contrast to the bright color of the middle region of the Al ribbons. The dark stripes correspond to the nano-trenches, whereas the bright stripes in the middle correspond to the Ge ribbons underneath the Al ribbons. We also used a focused ion beam (FIB) to cut individual resonators and expose their cross-sections for SEM imaging. A cross-sectional SEM image of a cut resonator in the lower right panel of Fig. 3b clearly shows that the designed nano-trench structures were successfully formed using the developed process.

### Trapping functionality of nano-trench structures

After device fabrication, we first tested the proposed passive molecule trapping functionality of the nano-trench structures. Three water-based solutions of different chemicals were prepared: l-proline (1 mg/mL), d-glucose (1 mg/mL), and regular table salt (mostly sodium chloride, 40 μg/mL). A small droplet (~1 μL) of each solution was dropped onto the device surface using a micro-pipette, which dried slowly under ambient conditions and eventually led to the precipitation of the dissolved chemical on the device surface. The concentrations of these solutions are high enough that the precipitated chemicals should completely fill most of the nano-trenches, which can be easily observed. Top-view optical microscopy images of the device structures with precipitated chemicals are shown in Figs. 3d–f. Compared with the bare device image in Fig. 3c, most of the precipitated chemicals accumulated near the edges of the Al ribbons, whereas the regions between adjacent Al ribbons were mostly clean. Although the optical images did not provide direct observation of chemicals inside the nano-trenches, they agreed with our expectation that the precipitation of dissolved molecules in an evaporating solution takes place mostly inside and near the nano-trenches. A video recording of the process of solution drying on the device surface (see Supplementary Information) also clearly shows that as the solution evaporated, it concentrated inside and near the nano-trenches, which eventually evaporated completely and led to precipitation of the dissolved chemical. In addition to trapping molecules in a solution, we found that such nano-trench structures can also effectively trap nano-particles in a gradually dried nano-particle suspension. We used a suspension of liposomes containing Cyanine 5 (Cy5) fluorescent dyes in our experimental demonstration. The Cy5-labeled liposomes have a diameter of ~100 nm. The fluorescent dyes allowed us to observe the spatial distribution of liposomes using a confocal microscope. To facilitate the fluorescence imaging of liposomes inside the nano-trenches, we replaced the aluminum ribbons with transparent photoresist ribbons (see Supplementary Fig. S2a). As shown in Supplementary Fig. S2b, c, the intensity of fluorescence was much higher in the nano-trench regions than in the other regions, such as between the Al ribbons (see Supplementary Information), which indicated that most of the liposomes were trapped in the nano-trenches.

### Characterization of device sensing performance

**Fig. 4:**
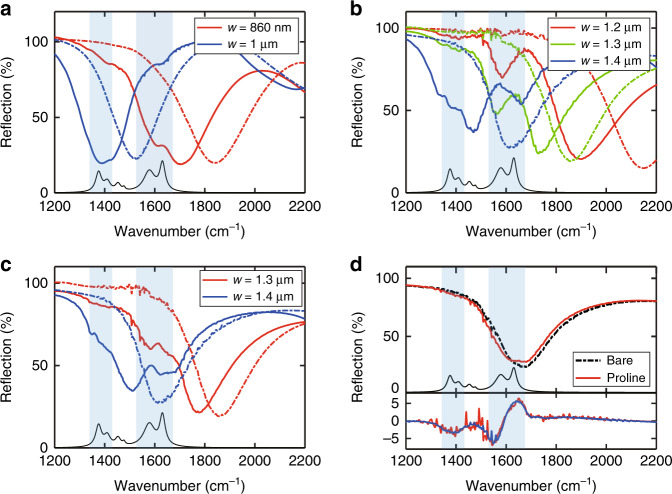
l-proline sensing results. **a**, **b** Reflection spectra of two sets of devices with **a** 200 nm wide nano-trenches and **b** 450 nm wide nano-trenches before (dot-dashed curves) and after (solid curves) introducing a 1 μL droplet of 10 μg/mL proline solution. The Al ribbon widths of the devices are specified in the legends. **c** Reflection spectra of two devices with 450 nm wide nano-trench, before (dot-dashed curves) and after (solid curves) introducing a 1 μL droplet of 1 μg/mL proline solution. **d** Upper panel: reflection spectra of the device with 450 nm wide nano-trenches and 1.4 μm-wide Al ribbons before (dot-dashed black curve) and after (solid red curve) introducing a 1 μL droplet of 0.2 μg/mL proline solution. Lower panel: the differential reflection spectra extracted from the spectra in the upper panel. The red (blue) curve is the differential spectrum before (after) the procedure for removing/reducing the interfering spectral features owing to water vapor absorption lines. Both differential spectra show clear spectral features associated with the proline absorption lines

To evaluate the sensing performance of our devices, we first used l-proline as the target analyte molecule and characterized the spectral responses of our SEIRA sensors to trace amounts of l-proline precipitated from low-concentration solutions. For each measurement, a droplet of ~1 μL of water-based proline solution with a certain concentration was dropped on the device surface and gradually dried under ambient conditions. Reflection spectra of the devices before and after introducing the analyte were measured with a Fourier transform infrared spectrometer (FTIR) connected to an infrared microscope (see “Materials and methods”). We started with a proline solution of 10 μg/mL (~87 μM) and applied it to two sets of devices with either 200 nm wide nano-trenches or 450 nm wide nano-trenches. The measured reflection spectra are shown in Fig. 4a, b. Compared with the reflection spectra of the bare devices (dashed lines), the reflection spectra of both sets of devices after proline was introduced show a significant peak frequency shift and clear spectral features associated with the proline absorption lines. As expected, such spectral features are stronger when the corresponding proline absorption lines are closer to the peak resonance frequency of the resonators. Overall, the set of devices with 450 nm wide nano-trenches produce considerably stronger spectral responses than those with 200 nm wide nano-trenches, which is consistent with our simulation results in Fig. 2. As proline has a relatively high solubility in water, the proline precipitate on the devices can be completely removed by rinsing with deionized water. This allowed us to conduct repeated proline sensing measurements using the same devices on proline solutions of various lower concentrations to explore the sensitivity limit of these devices. Figure 4c shows the reflection spectra of two devices with 1.3 μm- and 1.4 μm-wide Al ribbons and 450 nm wide nano-trenches in response to ~1 μL proline solution at a concentration of 1 μg/mL (~8.7 μM). The reflection spectrum change owing to the 1 μg/mL proline solution still has a considerable peak frequency shift and relatively strong spectral features associated with the proline absorption lines. Figure 4d upper panel shows the reflection spectrum change owing to ~1 μL proline solution at an even lower concentration of 0.2 μg/mL, which is smaller but nevertheless clearly visible. Note that the total amount of proline in one 1 μL droplet of 0.2 μg/mL proline solution is only 200 pg, and only a small fraction of this total amount was precipitated within the device area (~150 μm by 150 μm), from which the reflection spectra were measured. To better visualize the spectral response to such a trace amount of analyte molecules, we extracted the difference between the reflection spectrum of the device with the proline precipitate and that of the bare device, which is plotted in the lower panel of Fig. 4d. As these devices have zero transmission, the reflection change is essentially the negative of the absorption change. To extract the differential spectrum only due to the proline absorption lines, the reflection spectrum of the bare device was first redshifted so that the broad resonance in the two spectra overlapped across almost the entire spectral range, except around the proline absorption lines (see “Materials and methods” and Supplementary Fig. S3). This differential spectrum (the red curve in Fig. 4d lower panel) shows a clear feature with an asymmetric line shape near the two proline absorption lines at 1630 cm^−1^ and 1577 cm^−1^ and a peak-to-peak amplitude of ~10%, which is significantly above the noise level of the FTIR measurement (<1%). The observed line shape is also in good agreement with the simulated results in Fig. 2, although the two proline absorption lines were not individually resolved as in the simulation. Furthermore, another weaker (~3% amplitude) spectral feature associated with the proline absorption lines near 1450 cm^−1^ was also observed. The measurements were conducted under ambient conditions, and therefore, the spectra contain interference from water vapor absorption in the optical path within the spectral region of interest, which corresponds to the narrow peaks in the lower panel of Fig. 4d. However, as these water vapor absorption lines are narrow and at fixed wavelengths, they can be removed from (or significantly reduced in) the measured spectra with appropriate data processing. The blue curve in the lower panel of Fig. 4d is the differential reflection spectrum after performing a data processing procedure for removing the water vapor absorption lines (see “Materials and methods” and Supplementary Fig. S3). The spectral features associated with the proline absorption lines are much higher than the spectral noise and a few times larger than any residual water vapor absorption lines. According to the IUPAC definition of the limit of detection (LoD)^40^, the obtained spectral signal should be well above the LoD of these sensors using our measurement setup (see Supplementary Information). On the other hand, the LoD of our sensors can also be improved by optimizing our experimental setup. For example, the water vapor absorption lines can be removed or greatly reduced by purging the measurement system with nitrogen gas, and the open-path part of the experimental setup can be enclosed by opaque shields to reduce background radiation fluctuation.

**Fig. 5:**
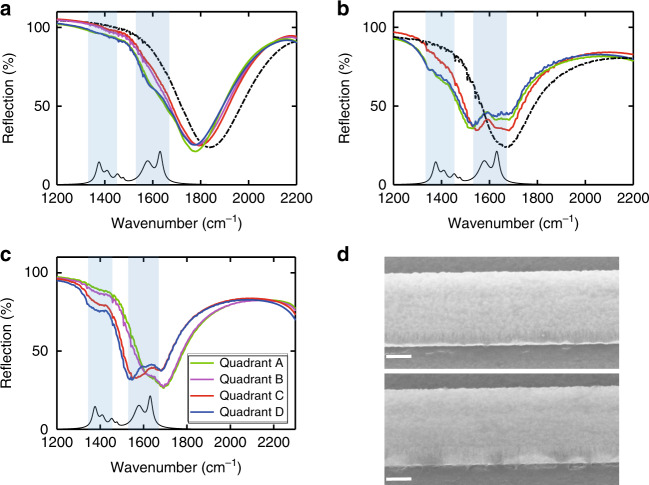
Reliability test of l-proline sensing. **a**, **b** Reflection spectra of repeated sensing experiments for 1 μg/mL proline solution by using a sensor with 200 nm wide nano-trenches in **a** and a sensor with 450 nm wide nano-trenches in **b**. The black dot-dashed curve in each graph is the reflection spectra of the bare device. **c** Reflection spectra measured at the four quadrants of the sensor in **b** after two 1 μL droplets of 0.2 μg/mL proline solution were applied. Each quadrant (A, B, C, or D) has an area of ~150 μm by 150 μm. **d** Representative SEM images showing the non-uniform proline precipitate distribution in quadrant B (top) and quadrant D (bottom). The SEM images are taken at a view angle of 45° of the device surface normal. The structures and color changes near the lower edge of the Al ribbon in each image correspond to the proline precipitate. The scale bar is 400 nm

To test the reliability of our devices for sensing such low-concentration analyte solutions, we chose several devices to repeat the same experimental process multiple times for each proline solution concentration. After each measurement, the device was thoroughly cleaned with deionized water to completely remove the proline precipitate, which was confirmed by measuring the reflection spectrum of the cleaned device before we applied a new droplet of solution for the next measurement. The reflection spectra of repeated sensing experiments for a 1 μg/mL proline solution using a device with 200 nm wide nano-trenches and another device with 450 nm wide nano-trenches are presented in Fig. 5a, b, respectively. Both devices produced an observable reflection spectrum change in each measurement. However, the amplitude of the spectral change varied from measurement to measurement. This spectral response variation across repetitions of a nominally identical experimental process is mainly owing to the distribution variation of precipitated proline across the device area, which is non-uniform and different each time. Comparing the spectral changes in Fig. 5a with those in Fig. 5b, we can see that the device with 450 nm wide nano-trenches consistently produced a significantly larger spectral response than the device with 200 nm wide nano-trenches and hence can more reliably sense trace amounts of proline precipitate despite the experimental variation. Figure 5c shows the reflection spectra measured from the four quadrants of the device with 450 nm wide nano-trenches (the total device area is 300 μm by 300 μm, and each quadrant is 150 μm by 150 μm) after two droplets of 0.2 μg/mL proline solution were introduced to the device surface (two droplets were used for this measurement to enhance the response signal). The spectra in quadrants C and D clearly exhibit a stronger response than those of quadrants A and B. This is again a result of the non-uniform distribution of the precipitated proline across the device surface, which can be seen from the SEM images in Fig. 5d taken from a view angle 45° off the vertical direction. The top and bottom SEM images in Fig. 5d represent the typical distribution of precipitated proline in quadrant B and quadrant D of the device in this particular experiment, respectively. These SEM images also clearly show that the proline precipitate was indeed in the nano-trenches, but it only occupied a small fraction of the nano-trench volume when the analyte solution was of such a low concentration.

**Fig. 6:**
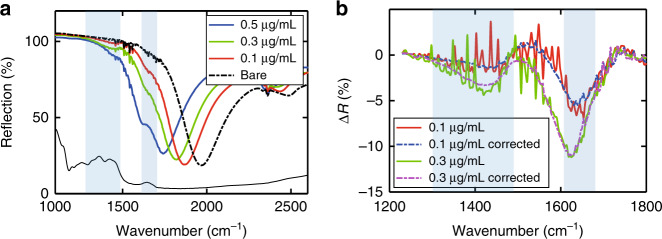
d-glucose sensing results. **a** Reflection spectra of a device before (dot-dashed curves) and after (solid curves) introducing a 1 μL droplet of glucose solution at three different concentrations. The black solid curve corresponds to the glucose absorption spectrum. **b** Extracted differential spectra from the data of the two lowest concentrations in **a**, both before and after applying the correction for the interfering spectral features due to the water absorption lines

As another example to demonstrate the sensing performance and versatility of our devices, we further conducted sensing of d-glucose in low-concentration solutions. Glucose has an important role in human metabolism, and monitoring the glucose concentration in blood is crucial for people with diabetes. Glucose has multiple absorption lines in the 1000 cm^−1^ to 1500 cm^−1^ spectral range, with several of them forming a relatively broad absorption band from 1330 cm^−1^ to 1460 cm^−1^ (see Fig. 6a)^21,41^. There is also a relatively weaker absorption line at ~1640 cm^−1^, which is due to adsorbed water, as glucose is hygroscopic. These glucose molecular absorption lines are within the spectral range of our SEIRA sensors developed for sensing proline. We prepared glucose solutions of various concentrations and followed the same experimental procedure to deliver droplets of the solutions to the device surface and characterized the spectral change due to the glucose precipitate. Figure 6a shows the spectral response of a device to a 1 μL droplet of glucose solutions at three different concentrations, i.e., 0.5 μg/mL (~2.8 μM), 0.3 μg/mL (~1.7 μM), and 0.1 μg/mL (~0.56 μM). The device produced a strong response to all three glucose concentrations. The spectral feature associated with the 1640 cm^−1^ absorption line was considerably stronger than that associated with the 1400 cm^−1^ absorption band, mainly because the resonance frequency of the device was closer to the 1640 cm^−1^ absorption line. The differential spectra extracted from the measurement results in Fig. 6a (both before and after applying the correction for the water vapor absorption lines) are shown in Fig. 6b. The spectral feature associated with the 1640 cm^−1^ absorption line reached an amplitude of ~10% at a 0.3 μg/mL concentration and ~5% at a 0.1 μg/mL concentration, whereas the 1400 cm^−1^ absorption band also led to a spectral feature with a different amplitude.

## Discussion

In terms of the total amount of analyte molecules contained in a solution droplet, our most sensitive devices reliably produced clear spectral responses to 200 pg of proline and 100 pg of glucose. However, as a 1 μL solution droplet typically covers a circular area with a diameter of ~2 mm on a device chip, whereas each sensor (resonator array) only occupies a square area of 300 μm by 300 μm, the actual amount of analyte molecules precipitated within the sensor area should be more than an order of magnitude smaller than the total amount contained in the solution droplet. Moreover, as the reflection spectra were measured from a 150 μm by 150 μm area within each resonator array, the number of analyte molecules involved in a spectrum measurement should be on average approximately a quarter of the amount within the entire sensor area. If we assume that on the macroscopic scale, the analyte molecules are distributed uniformly across the entire droplet area, then we can estimate that the spectral responses shown in Fig. 4d and 6c are owing to only ~1.4 pg of proline and ~0.7 pg of glucose, respectively. To put such sensitivity levels in another perspective, if these trace amounts of analyte molecules form a uniform thin film covering the measurement area, they would correspond to an ~0.5 Å thick proline film and an ~0.2 Å thick glucose film, respectively. Such thickness values are significantly less than those of the corresponding molecular monolayers. Therefore, the sensitivity of our current devices represents state-of-the-art SEIRA sensing, although the field enhancement in the hot spots of these nanophotonic resonators is orders of magnitude lower than that of some previously demonstrated sensor structures employing nanometric gaps^19–21^. The passive molecule trapping functionality of the nano-trench structures in our devices plays a crucial role in achieving such high sensitivity performance. By improving the measurement setup (such as using a purged environment to reduce water absorption) or adding a simple structure on the device surface to confine the droplet within the resonator array area, our current devices should be able to produce clear SEIRA responses to even lower solution concentrations. On the other hand, the current nanophotonic resonator designs can be optimized to achieve much larger field enhancement while preserving the molecule trapping functionality, which may lead to further improvement of the device sensitivity by orders of magnitude.

In summary, we experimentally demonstrated a proof-of-concept design of nanophotonic SEIRA sensors employing nanoscale structures that not only form photonic hot spots with large-field enhancement but also have the functionality of concentrating and trapping analyte molecules in an evaporating solution in hot spots, hence leading to significantly improved SEIRA sensing performance. The trapping mechanism requires no external energy source (i.e., passive) and is a result of the evaporation process of liquid in contact with the designed nano-structures and therefore is not limited to specific molecular species. We experimentally demonstrated that the trapping functionality of our devices applies to various molecular species and nano-particles, such as liposomes. To investigate the sensing performance of our SEIRA sensors, we used l-proline and d-glucose as target analyte molecules and achieved reliable sensing of precipitated analyte molecules with a mass down to ~1 pg, which corresponds to significantly less than one monolayer of analyte molecules when averaged over the entire measurement area. The sensitivity may be further improved by orders of magnitude with both improvements of the experimental setup and optimization of the device structure. The demonstrated SEIRA sensor design strategy can also be applied to other types of optical sensors. In addition to molecular sensing applications, such device structures can also be used for sensing and/or manipulating nanoscale objects, including exosomes, viruses, and quantum dots.

## Materials and methods

### Numerical simulation

Numerical simulations were carried out using commercial software (Lumerical FDTD Solution) that is based on the FDTD method. Owing to the translation invariance of the resonator structures along the Al ribbon direction, two-dimensional simulations were performed to design the resonator structures and investigate the spectral response to added analyte molecules. The relative permittivity of proline was derived from the measured absorption spectrum of a thermally sublimated proline thin film (see Supplementary Information Fig. S1).

### Device fabrication

A silicon substrate was cleaned with acetone/isopropyl alcohol (IPA) in an ultrasonic bath, followed by oxygen plasma cleaning. A 10 nm/200 nm thick Ti/Au film and a 200 nm thick Ge film were sequentially deposited on the silicon wafer using electron beam evaporation (Kurt J. Lesker). The samples were spin-coated with an ~250 nm thick layer of poly(methyl methacrylate) (PMMA, molecular weight 495 K, MicroChem, 495PMMA A4) and then a second layer of PMMA (molecular weight 950 K, MicroChem 950PMMA A4). The samples were baked at 180 °C for 2 minutes. The ribbon arrays were patterned using electron beam lithography at a 100 kV accelerating voltage, 10 nA beam current, and 1200 μC/cm^2^ exposure dose. The exposed PMMA was developed in a 1:3 solution mixture of methyl isobutyl ketone and IPA at room temperature for 60 seconds. Then, 5 nm Ti and 200 nm Al films were deposited with electron beam evaporation followed by a metal lift-off process in acetone, which formed Al ribbon arrays. An oxygen plasma cleaning step was conducted to fully remove the residual PMMA or other organic contaminants. Using the Al ribbons as hard masks, the Ge layer was partially etched by reactive ion etching (RIE) with CF_4_ gas plasma (flow rate 50 sccm, pressure 50 mTorr, power 100 W). The RIE etching process not only removed the Ge film between Al ribbons but also produced undercuts beneath the Al ribbons, which formed the designed nano-trenches. After every 20 seconds of RIE etching, SEM images of the samples were taken to determine whether the desired nano-trench length was achieved.

### Spectral measurement

The analyte solutions were prepared by first making relatively high-concentration solutions (1 mg/mL) and subsequently diluting them to various desired low concentrations. Approximately 1 μL of analyte solution was drawn from the solution container using a micro-pipette (adjustable volume range 0.5–5 μL) and dropped onto the chip surface, and occasionally, a rubber squeeze bulb was used to push the droplet to the chip area where the target devices were located. The chip was then left under ambient conditions for the droplet to fully evaporate. Reflection spectra were measured using FTIR (Bruker Vertex 70 v), which was connected to an infrared microscope with a reflective Cassegrain objective (NA = 0.4, ×15 magnification). A liquid nitrogen-cooled mercury cadmium telluride detector was used. The broadband infrared light emitted by the FTIR Globar passed a polarizer to obtain the polarization perpendicular to the Al ribbons before it was incident on the devices. The reflection spectra were measured from areas of the devices with a size of ~150 μm by 150 μm, which was controlled by an adjustable aperture in the optical path of the microscope. A bare gold mirror was used as the reference sample for all reflection spectral measurements. Each measurement was acquired at a scanner velocity of 40 kHz and a spectral resolution of 2 cm^−1^, and each spectrum was averaged over 500 scans (2-minute measurement time). All spectral measurements were carried out under ambient conditions at room temperature. After each set of measurements, the chip was immersed in deionized water for ~10 minutes to remove the analyte molecules, which was verified by a subsequent reflection spectral measurement of the cleaned device.

### Spectral data analysis

When the analyte solution concentration is relatively low, the broad resonance in the reflection spectrum of a device undergoes a moderate redshift while preserving its line shape (see, for example, Fig. 4d upper panel). Therefore, by applying a redshift to the reflection spectrum of the bare device accordingly, we could overlap the broad resonance in the reflection spectrum of the bare device to that of the device with a trace amount of analyte precipitate. The calculated difference between these two spectra is the differential spectrum, which is mainly due to the analyte molecules’ absorption lines (see, for example, Fig. 4d lower panel). However, this method of extracting the differential spectrum does not apply to relatively high-concentration analyte solutions because in those cases, the resonance in the reflection spectrum not only undergoes a larger redshift but also changes its line shape significantly (see, for example, Fig. 4b, c). Without further data processing, the extracted differential spectrum also contains significant interfering spectral features due to water vapor absorption lines. To remove or reduce these interfering spectral features due to the water vapor absorption lines, we performed the following data processing steps. We first obtained a smooth baseline fit (*R’*_bare_) to the measured reflection spectrum (*R*_bare_) of the bare device using a high-order polynomial function and then calculated the difference between *R*_bare_ and *R’*_bare_, i.e., δ*R*_bare_ = *R*_bare_ – *R’*_bare_. This extracted δ*R*_bare_ mainly consisted of narrow spectral features due to the water vapor absorption lines. As the measured reflection spectrum of the same device with the analyte precipitate (*R*_sens_) contained similar spectral features due to the water absorption lines, we subtracted δ*R*_bare_ from *R*_sens_ to obtain a reflection spectrum corrected for these water absorption lines, i.e., *R’*_sens_ = *R*_sens_ – δ*R*_bare_. Finally, we calculated the difference between *R’*_sens_ and the redshifted *R’*_bare_ to obtain the differential spectrum, which was mainly due to the analyte absorption lines, with much weaker spectral features associated with the water absorption lines (see, for example, Fig. 4d lower panel and Fig. 6b). The intermediate spectral results leading to the final differential spectra in Fig. 4d are included in Supplementary Fig. S3.

## Supplementary information

Supplementary Information Document

Supplementary Information Video
