# Supplementary material for: High-sensitivity nanophotonic sensors with passive trapping of analyte molecules in hot spots
Source: Light Sci Appl. 2021 Jan 5;10:5. doi: 10.1038/s41377-020-00449-7 (PMC7785746; doi:10.1038/s41377-020-00449-7)
Supplement: Supplementary file 1 — Supplementary Information Document [file 41377_2020_449_MOESM1_ESM.docx]

### Supplementary Information for

### High-Sensitivity Nanophotonic Sensors with Passive Trapping of Analyte Molecules in Hot Spots

**Xianglong Miao^1^, Lingyue Yan^2^, Yun Wu^2^, Peter Q. Liu^1^***

*1. Department of Electrical Engineering, University at Buffalo, The State University of New York, Buffalo, NY 14260, United States*

2. *Department of Biomedical Engineering, University at Buffalo, The State University of New York, Buffalo, NY 14260, United States*

*Author e-mail address: pqliu@buffalo.edu*

### Extracting the relative permittivity of proline from experimental data

We conducted IR reflection absorption spectroscopy measurement of a thin proline film on a gold mirror, and extracted the relative permittivity function of proline from the measured spectrum. The reflection spectrum$R\left( \theta\right)$ of a thin film (i.e. $t\ll\lambda$) on a gold mirror (close to a perfect mirror in IR) can be approximated by the formula:

$$\frac{R\left( \theta\right)}{R_{0}\left( \theta\right)}\approx1-4t\frac{\omega}{c}\frac{\sin^{2} \left( \theta\right)}{\cos^{2} \left( \theta\right)}Im\left\{ -\frac{1}{\varepsilon_{p}} \right\} (1)$$

where $\theta$ is the angle of incidence, $R_{0}\left( \theta\right)\approx1$ is the reflection spectrum of the bare gold mirror (close to a perfect mirror in IR),$t$ is the thickness of the thin film and $\varepsilon_{p}$is the complex relative permittivity of the thin film. In our experiment, the proline thin film was sublimated on the gold mirror by heating proline powder at 150 degree Celsius in vacuum. The thickness of the thin film is around 150 nm to 200 nm, which was measured using a profilometer. The reflection spectra were measured for both *p-*polarization and *s*-polarization of the incident light at 80° incident angle, which are plotted in Fig. S1a. Six absorption peaks of different strengths in the spectral region from 1300 cm^-1^ to 1700 cm^-1^ can be clearly observed in the reflection spectrum of the *p*-polarization, which are listed in Table S1. We modeled the relative permittivity function of proline using the Lorentz model and taking into account these six absorption lines, which is expressed by the following formula:

$$\varepsilon_{p}\left( \omega\right)= \varepsilon_{\infty}+ \sum_{j=1}^{6} \frac{S_{j}\omega_{0, j}^{2}}{\omega_{0, j}^{2}-\omega^{2}-i\omega\gamma_{j}} (2)$$

where $\omega_{0,j}$, $S_{j}$, $\gamma_{j}$ are the angular frequency, the oscillator strength, and the damping rate of the j-th absorption line, respectively. Assuming the non-dispersive component of the relative permittivity to be $\varepsilon_{\infty}=2.1$ (ref. 1), the Lorentz model parameters $S_{j}$ and $\gamma_{j}$ were extracted by fitting equation (1) to the experimental data, and the fitting results are listed in Table S1. The calculated relative permittivity function of proline using the fit parameters is plotted in Fig. S1b.

Table S1. Parameters for the proline relative permittivity model

| $\omega_{0,j} (cm^{-1})$ | 1375 | 1410 | 1453 | 1477 | 1577 | 1630 |
| --- | --- | --- | --- | --- | --- | --- |
| $\gamma_{j} (cm^{-1})$ | 28 | 30 | 26 | 12 | 55 | 26 |
| $S_{j}$ | 0.0081 | 0.004 | 0.0026 | 0.00045 | 0.0145 | 0.008 |

**
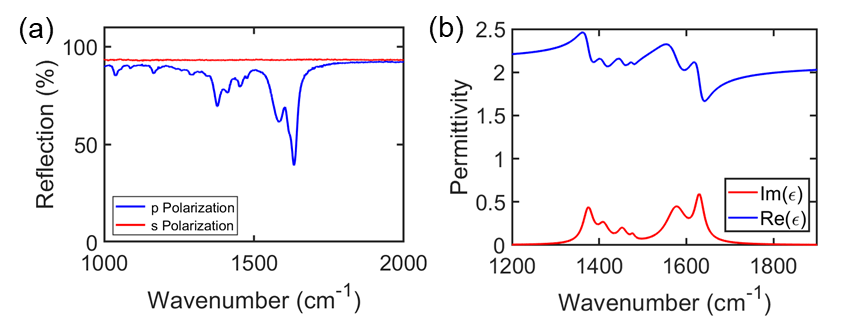
**

**Figure S1** **a** Reflection spectra of a thin proline film on a gold mirror at 80° incident angle, for both *p*- and *s*-polarizations. **b** Extracted relative permittivity of proline based on the Lorentz model.

### Preparation and fluorescence imaging of liposomes containing fluorescent dye molecules

The Cy5-labelled liposomes were prepared using the ethanol injection method. Briefly, a lipid mixture of 1,2-di-O-octadecenyl-3-trimethylammonium propane (DOTMA, Avanti Polar Lipids, 890898P), cholesterol (Sigma-Aldrich, C3405) and D-α-Tocopherol polyethylene glycol 1000 succinate (TPGS, Sigma-Aldrich, 57668-5G) was prepared at DOTMA:cholesterol:TPGS = 49.5:49.5:1 molar ratio in ethanol. Empty liposomes were prepared by injecting 1 part of the lipid mixture to 9 parts of 20 mM HEPES buffer. Then, Cy5 labelled oligonucleotides (Cy5-ODN, 5’-Cy5-GGCTAAATCGCTCCACCAAG-3’, Alpha DNA) were mixed with empty liposomes at the Cy5-ODN to lipid mass ratio of 1:10. The mixture of empty liposomes and Cy5-ODN was sonicated at room temperature for 10 minutes to form liposomes containing Cy5-ODN.

The device structures used for trapping liposomes are similar to our SEIRA sensors, except that the top Al ribbons were replace by photoresist (PR) ribbons for direct imaging of fluorescence emitted from the nano-trenches (see Fig. S2a). The liposome solution at Cy5-ODN concentration of 1 mg/mL was delivered to the device surface with the dip-coating method. The device was then left in ambient condition to dry. We used a Zeiss LSM 710 Confocal Microscope equipped with a Zeiss In Tune laser (emission wavelength can be tuned in the range of 488 nm to 640 nm) and spectral detectors. As this is an inverted microscope system, the device was placed with the top surface facing down onto a cover glass. The reflection image of the device structure (Fig. S2b) was taken with the excitation wavelength set to 488 nm and the detection wavelength range set to 506 nm, while the fluorescence image of the Cy5-labelled liposomes deposited on the device (Fig. S2c) was taken with the excitation wavelength set to 640 nm and the emission wavelength range set to 707 nm.

**
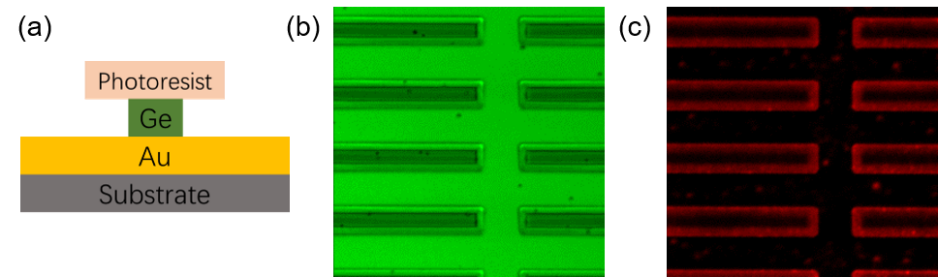
**

**Figure S2** **a** Schematic of the modified device structure used for liposomes trapping and fluorescence imaging. **b** Reflection image of the device structure using an illumination at 488 nm wavelength. The Photoresist ribbons, the underlying Ge ribbons and the nano-trenches were clearly observed. **c** Fluorescence image of the same device area in **b**. The excitation wavelength was 640 nm, and the emission wavelength was 707 nm.

**Procedure for extracting the differential reflection spectrum**

Figure S3 shows the spectra of the intermediate steps of the procedure for extracting the differential spectra (described in the “Materials and methods” section in the main text) in Figure 4d lower panel in the main text. Specifically, Figure S3a shows the red-shifted reflection spectrum of the bare device which overlaps well with the reflection spectrum of the same device with the proline precipitate from the 0.2 μg/mL solution, whereas their difference corresponds to the red differential spectrum plotted in Figure 4d lower panel; Figure S3b shows the polynomial fit (*R’*_bare_) to the reflection spectrum of the bare device (*R*_bare_) across the spectral range between 1200 cm^-1^ and 2000 cm^-1^, and the difference between the measured and the fit spectra, i.e. δ*R*_bare_ = *R*_bare_ – *R’*_bare_ , which mainly consists of spectral features due to the water absorption lines; Figure S3c shows the reflection spectrum of the device with the proline precipitate (*R*_sens_) and the corrected spectrum after subtracting δ*R*_bare_ (i.e. *R’*_sens_ = *R*_sens_ – δ*R*_bare_) to remove/reduce the interfering water absorption lines; Figure S3d shows the corrected reflection spectrum of the device with the proline precipitate (*R’*_sens_) and the red-shifted *R’*_bare_ , and their difference corresponds to the blue differential spectrum plotted in Figure 4d lower panel.

**
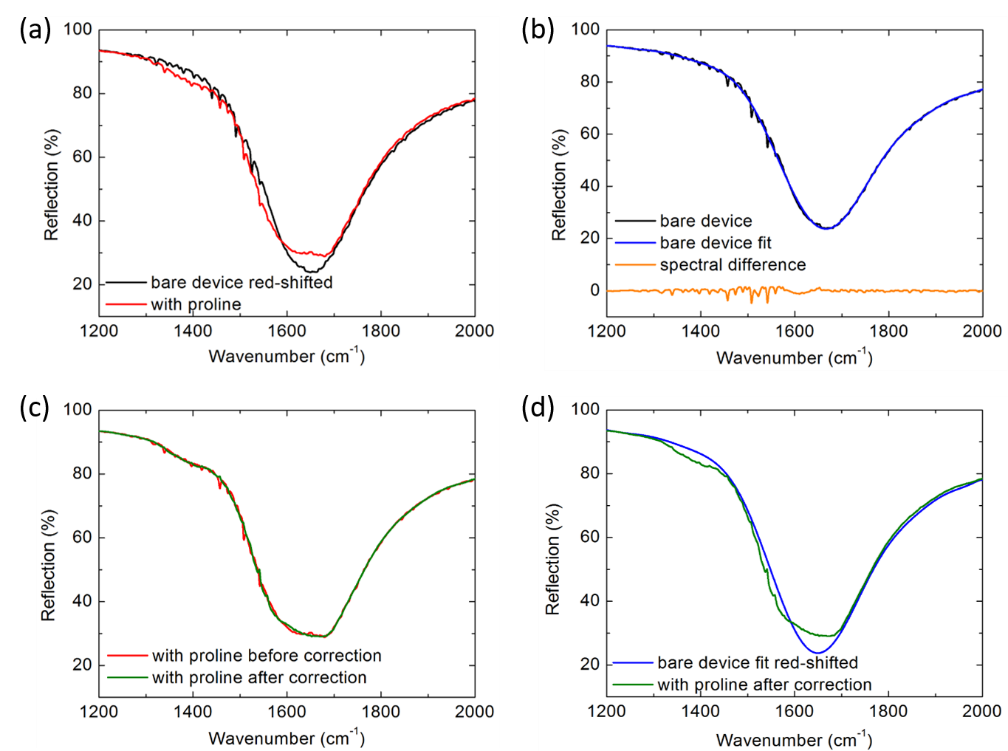
**

**Figure S3 a** Reflection spectrum of the bare device (red-shifted) and that of the same device with the proline precipitate. **b** Reflection spectrum of the bare device and its polynomial fit, as well as the difference between them. **c** Reflection spectra of the device with the proline precipitate before and after applying the correction for the water vapor absorption lines. **d** Reflection spectrum of the device with the proline precipitate after correction and the red-shifted polynomial fit of the bare device reflection spectrum.

**Limit of detection (LoD) analysis**

According to the definition of LoD by the International Union of Pure and Applied Chemistry (IUPAC), the LoD expressed as a concentration *c*_L_ is derived from the smallest signal *x*_L_ that can be detected with reasonable certainty.^2^ To ensure a relatively high certainty, the convention is to set the LoD threshold signal as $x_{L}=\bar{x}_{B}+3s_{B}$, where $\bar{x}_{B}$ is the mean value of repeated blank measurements (i.e. without analyte), and $s_{B}$ is the standard deviation of such blank measurements. In order to characterize the standard deviation of the blank measurements (i.e. reflection spectra of bare devices) using our measurement setup and parameter settings, we conducted 10 independent measurements of the reflection spectrum of a bare device sequentially, which are shown in Figure S4a. The standard deviation of these spectral measurements was then calculated at each frequency point, which is plotted in Figure S4b. Clearly, in the entire spectral range from 1000 cm^-1^ to 3000 cm^-1^, the standard deviation remains below 1%. Therefore, in our experiments, any spectral change around the absorption lines of an introduced analyte with more than 3% peak difference can be considered with a high confidence level as being above the LoD threshold signal. The fact that the standard deviation spectrum in Figure S4d has a similar shape as the reflection spectrum of the device in Figure S4a suggests that the dominant noise source may be the fluctuation of background radiation incident on the device. Therefore, by enclosing the entire optical path of our measurement setup with radiation shields (which is not the case currently) may further reduce the blank measurement fluctuation and improve the LoD.


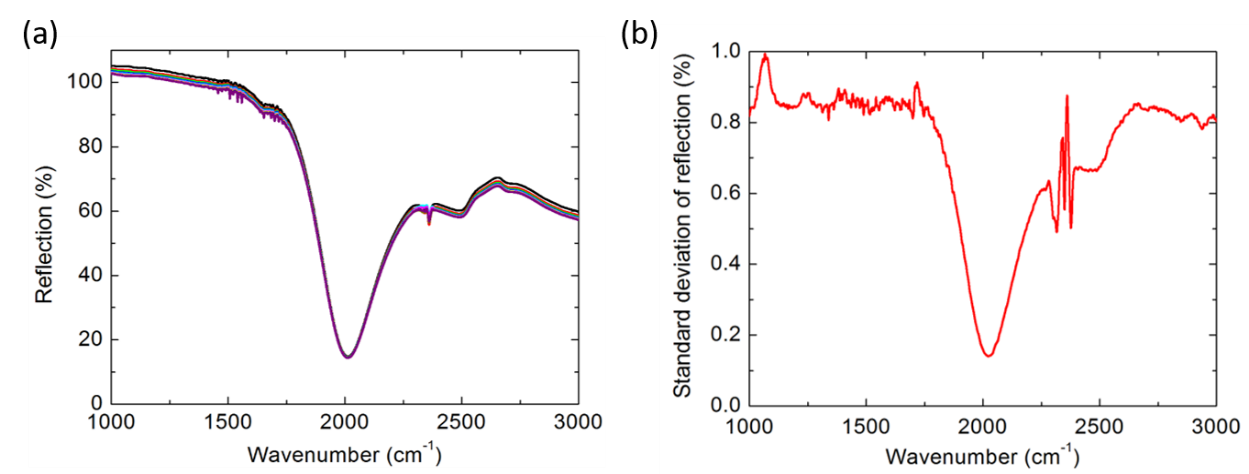


**Figure S4 a** Ten sequential and independent measurements of the reflection spectrum of a bare device using the same settings as the sensing experiments. **b** Calculated standard deviation of the reflection spectra plotted in **a**.

### Supplementary movie file

The movie shows process of a relatively high-concentration proline solution (1 mg/mL) drying on the device surface and leading to proline precipitation.

### Reference

1. Rodrigo, D. et al. Mid-infrared plasmonic biosensing with graphene. *Science* **349**, 165-168 (2015**)**.
2. Long, G. L. & Winefordner, J. D. Limit of detection. A closer look at the IUPAC definition. *Anal. Chem.* **55**, 712–724 (1983).
